# Integrating internationally qualified nurses: a qualitative exploration of nurse managers’ influence from nurses’ experiences

**DOI:** 10.1186/s12912-025-02875-7

**Published:** 2025-02-28

**Authors:** Catharina Roth, Amanda Breckner, Katja Krug, Cornelia Mahler, Michel Wensing, Sarah Berger

**Affiliations:** 1 Department of General Practice and Health Services Research, University Hospital Heidelberg, Marsilius Arcades, West Tower, Im Neuenheimer Feld 130.3, 69120 Heidelberg, Germany; 2 Department of Nursing Science, University Hospital Tuebingen, Hoppe-Seyler-Str. 9, 72076 Tuebingen, Germany; 3 Medical Faculty, Heidelberg University, Ruprecht-Karls-University Heidelberg, Grabengasse 1, 60117 Heidelberg, Germany; 4 Department of Nursing, University of Otago-Christchurch Campus, 2 Riccarton Ave, 9140 Christchurch, New Zealand

**Keywords:** Workplace integration, Internationally qualified nurses, Migration, Germany, Interview study, Organisational role, nursing management

## Abstract

**Background:**

Healthcare systems globally are confronted with a shortage of nurses. Various strategies to address this have been applied, including active recruitment of internationally qualified nurses. Nurse managers may have a central role in supporting workplace integration. This study aimed to explore how domestically qualified nurses and internationally qualified nurses viewed the role of nursing management and its impact on workplace integration.

**Method:**

Semi-structured interviews with 21 domestically and 14 internationally qualified nurses were conducted. Nurses were selected using purposive sampling. Snowball sampling was applied to reach a sufficient sample size. Interviews were pseudonymized and transcribed. Transcripts were coded according to Qualitative Content Analysis with data structured into themes and subthemes.

**Results:**

Five key themes related to impact of nursing management on workplace integration were identified: (a) Appropriate Placement, (b) Recruitment Process, (c) Leadership Support, (d) Workforce Shortage, and (e) Additional Burden/ Increased Workload. Active support by nursing leadership and the opportunity for shared-decision making was seen as a key factor. Increased workload, additional time and resources requirements associated with orientation of internationally qualified nurses and pressures from staff shortages were highlighted as hindrances. Inappropriate placement of internationally qualified nurses was perceived as key hindrance that could be addressed by nursing management. An imbalanced ratio between domestically and internationally qualified nurses was perceived as challenging by domestically qualified nurses.

**Conclusion:**

Integration of internationally qualified nurses to clinical practice brings several challenges that may be positively impacted by nursing management through appropriate placement of internationally qualified nurses, supportive nurse managers and adequate preparation of domestically qualified nurse mentors/preceptor.

**Implications for practice:**

Nurse managers should ensure that internationally qualified nurses’ work experience matches local clinical unit vacancies before hiring them. Peer support is a supportive factor for internationally qualified nurses. Nurse managers should find a balanced ratio between internationally and domestically qualified nurses in the roster. Introducing mentors or preceptors at ward level may be a strategy decrease work-related stress in both nursing groups.

**Registration number:**

The study has been prospectively registered (27 June 2019) at the German Clinical Trial Register (DRKS00017465).

**Supplementary Information:**

The online version contains supplementary material available at 10.1186/s12912-025-02875-7.

## Background

The global population is aging, global life expectancy at birth increased by more than six years between 2000 and 2019 [1]. This demographic change is also evident in Germany. According to the German Federal Statistical Office, there is a gradual decline in young people while the number of people over the age of 66 is increasing (20% of the overall population of Germany is older than 66). It is anticipated that the number of people needing long-term care in Germany will increase by 37% by 2055. Life expectancy at birth in Germany is currently 81.7 years [1].

This demographic pattern is also reflected in the healthcare workforce [2]. Healthcare professionals are not only ageing reflecting the rest of the population, but those entering the healthcare workforce also declining in numbers [3–5], which means a parallel decrease workforce supply while the demand for healthcare increases. In Belgium, Germany and New Zealand, for example, the number of nurses had declined by 10% up to 2011 and by 15% up to 2021 [6]. According to the World Health Organisation (WHO), nurses and midwives now account for 50% of the current global shortage of healthcare workers, which is forecasted to reach 9 million by 2030 [7]. A gap in the nursing workforce left by the retiring baby boomer generation is coinciding with a shrinking pool of younger people entering the (nursing) workforce [3]. Strategies to address the increasing demand for healthcare services by an aging population, which is dovetailing with shortages of healthcare professionals, need to be implemented if we are to reach the Sustainable Development Goal 3 on health and well-being.

Germany has already applied several strategies to address the increased demand for healthcare services and the decreasing supply of healthcare professionals, particular nurses. For example, measures to improve retention and working conditions [8], expand the recruitment base [8], and target returners to the nursing workforce [8] have been implemented. In June 2019, Germany launched its initiative “Konzentrierte Aktion Pflege [Focused Action for Nursing]”. This initiative included goals and concrete measures to improve working conditions for nurses and nursing aides and to update the nursing training curriculum. The specific objectives of the initiative are: improve working conditions, expand the recruitment base, attract new trainees, target nurses who have left the nursing profession to return, improve remuneration, digitalisation to reduce workload, and significantly, recruitment of internationally qualified nurses [9, 10]. The recruitment of internationally qualified nurses (IQNs) from abroad is not a new phenomenon in Germany. Nurses from South Korea and the Philippines came to Germany as early as in the 1960s and 1970s as part of a post war economic boom [11]. Currently, however, the recruitment of nurses is based on the changed framework conditions of an immigration policy that is politically motivated and designed to attract qualified nurses from abroad, while competing with other European countries such as Italy, the United Kingdom, Austria, Denmark and Switzerland doing the same [12, 13].

In order to provide effective and integrated care to patients, active coordination and organisation within and between hospitals is necessary. A structured approach to workforce management is essential to ensure that medical, nursing and allied health services develop and work efficiently [14]. Change is a common experience in healthcare settings. Staff, patients and visitors change [15], leadership, models of care, workforce are reshaped [16] and new technologies are introduced or retired [17]. The recruitment of IQNs and their integration into the German nursing workforce is creating significant changes in workforce and team structures and processes. IQNs not only add different cultural values and views to the organisation but may also share different professional experience and educational background based on the country in which they were trained [18]. The approach by the receiving organisation to workplace integration of IQNs into the nursing workforce and the healthcare setting is fundamental to its success.

Nurse managers play a central role in addressing workplace integration needs as they are responsible for overseeing the operation of individual wards, and for providing direct supervision and support for nurses directly involved in patient care [19–21]. Cummings et al. [22] conducted a systematic review and reported a strong link between leadership and management behaviours and various nurse outcomes, such as productivity and effectiveness, as well as job satisfaction and retention. Evidence suggests that nursing leadership behaviours influence nurses’ ability to contribute to organizational goals [23]. Moreover, leadership styles that support staff needs reduces likelihood of burnout by encouraging nurses to evaluate their work environment as challenging rather than overwhelming [24, 25]. Further factors that enable successful workplace integration included motivated local nursing team, structured orientation programs, preceptorship, and additional practical skills training and language training [18]. The expectations of IQNs and the reality they are confronted with after migration may also influence the success of integration. Failure to fulfil expectations may lead to IQNs leaving the profession in the long term [26, 27]. Unfulfilled expectations are also one of main reasons why DQNs leave the profession after a few years in clinical practice [28, 29].

Recruiting nurses from abroad to address the shortage of qualified nurses is only economically viable in the long term if workplace integration is managed and organised successfully. In the context of workplace integration of IQNs, experiences of domestically qualified nurses (DQNs) directly involved in the process are of particular interest. However, integration is a two-way process, so the perceptions of IQNs are also important to build a comprehensive understanding. Therefore, the aim of this study was to explore how domestically and internationally qualified nurses experienced and evaluated the role of their nursing management and their healthcare organisation during workplace integration.

## Methods

### Study design

Within an overarching research initiative “Nurse Migration Project” (in the Department of General Practice and Health Services Research of the University Hospital Heidelberg, Germany focused on generating in-depth knowledge on the experiences of both DQNs and IQNs during workforce integration) a qualitative study was undertaken with regards to the perceived impact of the role of nursing management on workplace integration by DQNs and IQNs. In this study, the methodological approach of qualitative research was applied due to the explorative nature of the research question *How do nurse managers influence workplace integration of internationally qualified nurses*. Phenomenology is a discipline concerned with exploring the experiences and perspectives of individuals who are directly influenced by a social phenomena. It was therefore considered as appropriate as an underpinning theoretical framework in order to answer the research question [30]. The aim of this approach was to explore the perspectives of domestically and internationally qualified nurses, taking social structures into account. Thus, the perspectives of those affected by recruitment and integration of IQNs were the centre of the research interest. Two studies (exploring different but related research questions) have already been published as part of the project [18, 29].

### Study setting

Four executive Directors of Nursing from four different German hospitals were approached and agreed to conduction of this study at their institutions; (a) Centre 1 (1.600 beds, 2601 nurses) a university hospital in the South of Germany that provides tertiary level healthcare, (b) Centre 2 (319 beds, 200 nurses) specialty thoracic hospital in the South of Germany, (c) Centre 3 (234 beds, 280 nurses) a regional public hospital, and (d) Centre 4 (200 beds, 200 nurses) a regional public hospital.

### Participants

The following eligibility criteria had to be fulfilled by DQNs and IQNs in order to participate:

- Domestically qualified nurses (DQNs) had to have graduated from a German nursing training program and therefore obtained a recognised qualification in nursing in Germany (e.g., state-qualified nurse).
- Internationally qualified nurses (IQNs) had to (a) have graduated from an international nursing training program and therefore obtained a recognised qualification in nursing in another country (outside of Germany either within the European Union or elsewhere internationally) and (b) work as nurse in the receiving country Germany. Elsewise they had to have obtained a recognised qualification in nursing in another country and have not yet obtained a (full) registration to practice as qualified nurse in the receiving country Germany. IQNs who had not yet obtained a (full) registration as qualified nurse usually were employed as nursing assistants.

Both DQNs and IQNs were required to be at least 18 years old, to have a contract with the involved hospitals and have a reasonable command of the German and/or English languages (written and spoken). Nurses from different general and specialty practice settings were invited (e.g. general wards, intermediate care units, and intensive care units) in order to maximise participant diversity. No minimum employment working hours was set. Student nurses, other healthcare professionals (e.g. physicians, physiotherapists, nurse aids), or domestically and internationally qualified nurses who did not consent to participate were excluded from the study.

### Sampling and recruitment process

The principle of purposive sampling was applied in this study. Different sampling and recruitment methods were applied at the four hospitals based on preferences of the approving bodies.

A primary contact person (appointed by the Director of Nursing) introduced the study and its purpose to the nursing managers of eligible wards at **Centre 1**. **At Centre 2**, a meeting was organised by the Director of Nursing in order to inform nursing managers of eligible wards about the study. In **Centres 1 and 2**, nursing managers were responsible for distributing information on the study to eligible nurses. Nursing managers of eligible wards were responsible to identify nurses within their nursing teams who met the inclusion criteria. In total, 130 DQNs and 96 IQNs met the inclusion criteria and were initially invited to participate at **Centre 1 and Centre 2** combined. Each nurse received an envelope with informational materials, including an invitation to participate in the study, an information sheet, an informed consent form, and a return envelope. The folder also contained contact information of the research team. Nurses who decided to participate in the study were asked to contact the research team directly via email or phone.

The Director of Nursing of **Centres 3 and 4** informed the nursing managers of eligible wards about the study, who in turn informed their nursing teams and decided collaboratively with the team who could potentially want to participate in an interview. These nurses also received the information material, including the invitation to participate in an interview, an information sheet, a consent form, and the response envelope. In total, five DQNs and one IQNs were willing to participate and were initially invited to take part in an interview at **Centre 3 and Centre 4** combined. The Director of Nursing and a research team member made appointments for interviews with the interested nurses.

Reminders were sent to each hospital’s Director of Nursing to increase response rate at four and six weeks after the initial distribution of the study material. In addition, nursing managers of wards were asked to promote the research project and encourage eligible nurses at regular team meetings. In addition, snowball sampling as a method within purposive sampling was applied due to difficulties in accessing a sufficiently large sample as recruitment partially took place during the first year of the COVID-19 global pandemic. Nurses who participated in interviews were encouraged to share the study invitation with colleagues. Snowball sampling, also known as network sampling, can be helpful if study participants belong to a group that is difficult to recruit or reach. Usually, recruitment begins with some study participants. These initial participants are used to identify and recruit additional participants [31, 32].

**Fig. 1:**
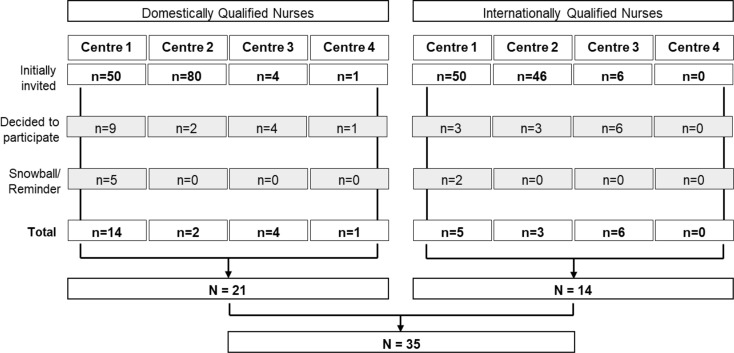
Sampling procedure base on a nonprobability sampling technique for DQNs and IQNs

In total, 135 domestically qualified and 102 internationally qualified nurses were invited to participate in an interview. At **Centre 1**, 50 DQNs were initially invited to participate in an interview, nine contacted the research team and proceeded to interview. Via the snowball sampling technique five further DQNs were recruited (*n*=14). In addition, 50 IQNs were invited to participate, three agreed and encouraged two more via snowball sampling (*n*=5). At **Centre 2**, 80 DQNs were initially invited to participate in an interview, two nurses proceeded to interview (*n*=2), no other nurse was recruited using snowball sampling. In total 46 IQNs were initial invited at **Centre 2**, three agreed to participate in an interview (*n*=3), no other nurse was recruited using snowball sampling. At **Centre 3**, four DQNs and six IQNs decided to participate, at **Centre 4**, only one DQN participated due to the SARS-CoV-2 pandemic and the increased measures to contain the spread of the virus (*n*=1). In total nineteen interviews were conducted at **Centre 1**, ten at **Centre 3**, five at **Centre 2**, and one at **Centre 4**. In all centres combined, a total of 21 interviews with DQNs and 14 interviews with IQNs were conducted (Fig. 1). No nurses who initially agreed to participate withdrew their consent. Seven of the participating nurses (employed at **Centre 1** and **Centre 2**) were known to the researcher through clinical work.

### Data collection

Semi-structured individual face-to-face or telephone interviews were conducted by a female health services researcher with a background in nursing and public health (CR) between September 2019 and August 2020. Data collection was interrupted and prolonged due to the COVID-19 global pandemic.

The first author (CR) initially developed the interview guide based on a literature review. The initial version was discussed with other researchers in a qualitative research colloquium at the Department of General Practice and Health Services Research, University Hospital Heidelberg. Adaptions to the interview guide were made based on feedback and recommendations from the colloquium participants (other health services researchers and sociologists). The interview questions were designed to be open-ended. Interview questions addressed experiences related to workplace integration of IQNs from the perspective of DQNs and IQNs. The interview guide was developed to provide data regarding several research questions in the “Nurse Migration” project. Accordingly, the interview guide was also used in previously published studies [18, 29]. The English version of the interview guide is attached as supplementary file 1.

Face-to-face interviews were either conducted in a separate and quiet room on the ward or at the Department of General Practice and Health Services Research, University Hospital Heidelberg. Participants that had elected to take part in a telephone interview were called by the interviewer. Most of the interviews took place before or after the participants shifts. Interviews with DQNs and IQNs were conducted simultaneously. Interviews were digitally recorded, pseudonymised and transcribed verbatim by the first author of this study and a research assistant. Each interview transcript was reviewed whilst listening to the digital recording to ensure accuracy. The transcription software “f4transkript” was used to transcribe the interviews. In addition to the interviews, information such as age, gender, work experience, and place of work were collected. Only the interview participant and the interviewer were present during the interview. No additional field notes were taken during or after the interview.

### Data analysis

**Fig. 2:**
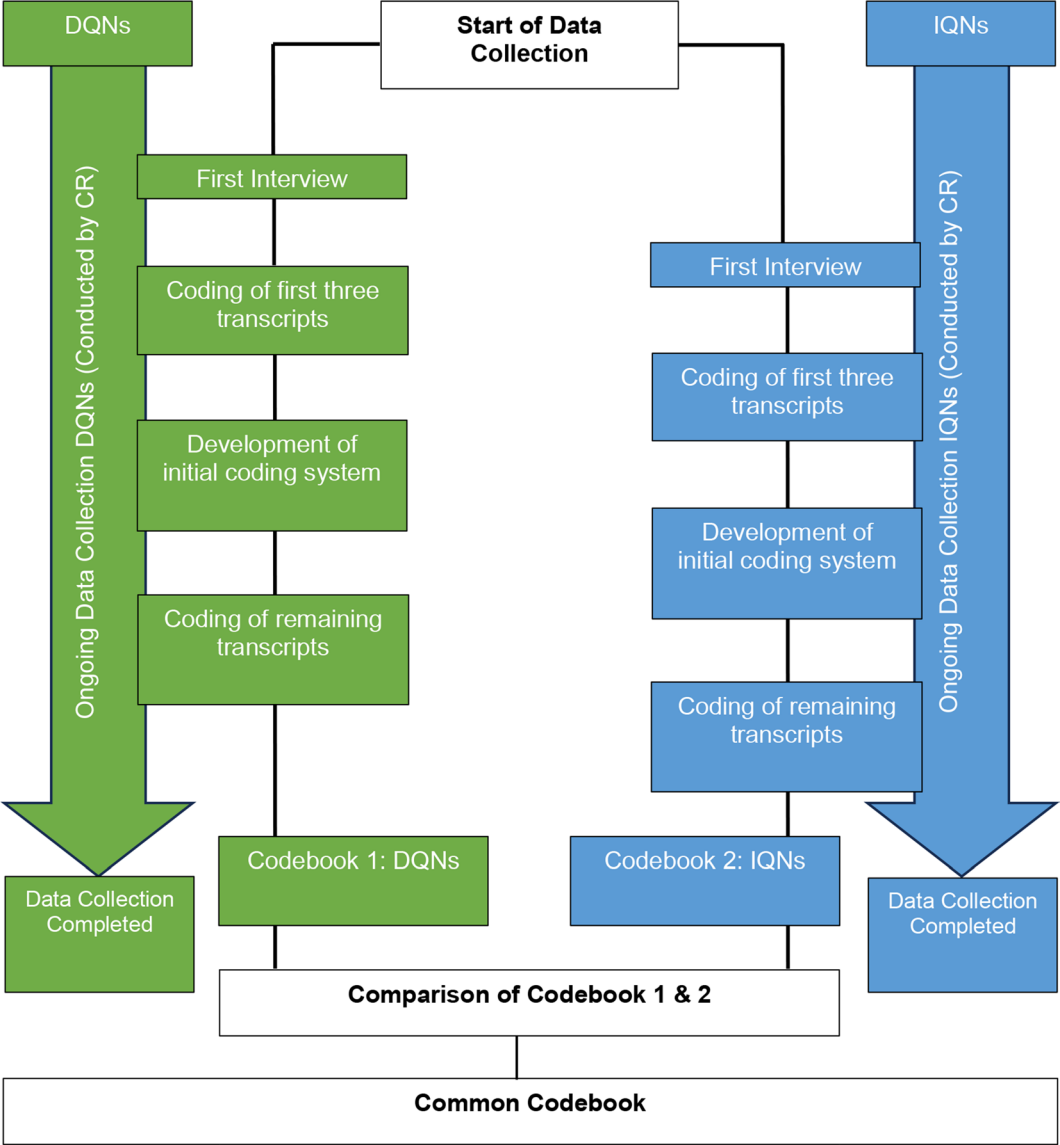
Representation of the data collection and analysis process applied in this study. Interviews with both study groups were conducted only by CR. Interviews with DQNs and IQNs were conducted simultaneously. The first interview was conducted with a DQN, the last one with a IQN. The transcription of the interviews was carried out by CR and a research assistant. All interview transcripts from both study groups were coded and analysed by CR and AB. Everything shown in green refers to DQNs; everything shown in blue refers to IQNs

Interview data was analysed according to Qualitative Content Analysis [33] by two female health services researcher (CR and AB). The data analysis process started during the early stages of data collection. Data sets were analysed the two coders (CR and AB). Figure 2 shows the data collection and analysis process.

**Step 1**: All interview transcripts (DQNs and IQNs) were formatted uniformly to improve readability. The first transcripts of both groups were read and essential text passages were marked by CR and AB (data coders). **Step 2**: CR and AB then familiarised themselves with both data sets (Set 1 DQNs and Set 2 IQNs) by reading further transcripts. Transcripts of the first three interviews of both data sets were coded independently by CR and AB. Constructed codes were discussed, and an initial coding system was developed by consensus for both data sets separately. **Step 3**: Each interview transcript was then coded line-by-line by CR and AB **Step 4**: In further discussions, each coded interview transcript was then compared against the coding framework by CR and AB. Disagreements about codes were resolved in consensus discussions with CR, AB and SB (senior health services researcher with a background in nursing). Central themes were developed based on the codes and sub-themes from the content analysis. Both researchers analysed all interview transcripts using the same methodology. **Step 5**: The final coding system (codebooks) included codes, subthemes, themes, and illustrative quotes. This analysis procedure was applied to both data sets (DQNs and IQNs) separately, resulting in two separate codebooks, including several similar subthemes and themes. The final codebooks were discussed between CR, AB, and SB to ensure consensus as part of the quality management process for qualitative data analysis. Each interview transcript was analysed using the same method. This analysis resulted in two different codebooks (Codebook 1: DQNs and Codebook 2: IQNs). During this analysis, ideas emerged regarding potential agreeing and disagreeing statements between DQNs and IQNs. **Step 6**: Both codebooks were scanned in detail to explore agreeing and disagreeing statements. **Step 7**: A table was created that included statements made by either or only one group. This table consisted of the initial codes, subthemes and themes developed during the first analysis (Step 1–5). **Step 8**: Based on the initially developed codebooks, these statements were structured into common subthemes and themes. **Step 9**: The separately presented findings of both study groups (Codebooks) of the first analysis steps, including the raw data, were scanned again and compared to the table that included deductively developed common subthemes and themes to ensure that no data was lost during this analysis step. The final coding framework including themes and sub-themes and illustrative quotes was discussed between the three researchers (CR, AB, and SB) to ensure consensus Fig. . 1). Interview data were analysed using MAXQDA, version 2020.1, a computer-assisted quality data management software [34].

### Data protection

Interview data collected in this study was assigned a unique STUDY ID number for each participant. Personal identifying information is stored in a separate database from the study data that was used for analysis. There is a code sheet/key linking participants’ personal identifying information to the unique study ID code. Only specific research team members had access to this code sheet. All databases used for analysis were de-linked from personal identifying information using the STUDY ID. Data was stored pseudonymised in a secure location, with the key to personal identifiers in a separate place. All data collected in this study was protected by the EU General Data Protection Regulation (Regulation (EU) 2016/679) as well as the German Federal Data Protection Act (Bundesdatenschutzgesetz (BDSG)).

### Management of data quality

In order to enhance the credibility of results the following steps were applied: using more than one data coder during data analysis (CR and AB) as well as consensus discussions between the two coders (CR and AB) and a senior researcher (SB). Confirmability was established by developing an audit trail of the research process. Transferability was ensured by describing the setting of data collection as precisely as possible. Dependability was established by the following steps: peer debriefing (colloquium with colleagues) and external audit (qualitative research colloquium). During the research colloquiums, the process of data collection, data analysis and preliminary findings were discussed. However, participants of both colloquiums were given no insight into raw data adhering to data protection regulations.

### Ethical considerations

The study was approved by the Medical Ethics Committee of the Medical Faculty of Heidelberg University (S-367/2019). The staff council of each hospital also gave permission for the study to be undertaken on their site. Written informed consent was provided prior commitment to the interviews by each participant. Confidentially was ensured for each participant by assigning a pseudonym. Only specific members of the research team had access to the pseudonymisation key and the associated personal data. This was explained in detail in the information leaflet that was handed out to each participant. Additionally, participants were assured that they could withdraw their consent to take part at any time. Personal data that had not yet been included in the data analysis or that had been anonymised could be deleted on request. Research conducted in this study was performed in accordance with the Declaration of Helsinki [35]. The study was reported according to the Consolidated Criteria for Reporting Qualitative Studies (COREQ) checklist for qualitative research [36].

## Results

A total of thirty-five qualified nurses were included in the study, 21 DQNs and 14 IQNs. Most nurses were employed at Centre 1 (54.3%). Twenty-one interviews were conducted face to face (60%) and fourteen by telephone (40%). The mean interview duration was 21 min (range 8–41 min).

The oldest nurse that participated was 62 years old, the youngest nurse was 22 years. The majority of nurses identified as female (74.3%), and had on average 16 years work experience in Germany. They were employed in different departments but mainly on normal wards (74.3%) (Table 1). The top country of origin of IQNs was the Philippines (35.7%), followed by India (14.3%), Italy (14.3%), and Romania (14.3%).

**Table 1:** Participants’ characteristics

*n* (%)	DQN* n = 21 (60.0)	IQN** n = 14 (40.0)	Total *N* = 35 (100.0)
**Study Centre**	*n* (%)		
Centre 1	14 (66.7)	5 (35.7)	19 (54.3)
Centre 2	2 (9.5)	3 (21.4)	5 (14.2)
Centre 3	4 (19.0)	6 (42.9)	10 (28.6)
Centre 4	1 (4.8)	0	1 (2.9)
**Interview Method**	**n (%)**		
Face to Face Interviews	13 (61.9)	8 (38.1)	21 (60.0)
Telephone Interviews	8 (57.1)	6 (42.9)	14 (40.0)
**Age in years**	**Mean (SD)**		
	40.4 (11.4) Min 22 Max 60	33.1 (10.4) Min 24 Max 62	37 (11.3) Min 22 Max 62
**Gender**	**n (%)**		
Female	17 (81.0)	9 (64.3)	26 (74.3)
Male	4 (19.0)	5 (35.7)	9 (25.7)
**Work experience in years**	**Mean (SD)**		
in Germany	19.5 (12.8) Min 0 Max 44	10.5 (8.3) Min 1 Max 28	16 (11.7) Min 0 Max 44
**Position**	**n (%)**		
State-Qualified-Nurse	14 (66.7)	13 (92.9)	27 (77.1)
Head Nurse	7 (33.3)	1 (7.1)	8 (22.9)
**Clinic**	**n (%)**		
Internal Medicine	5 (23.8)	3 (21.4)	8 (22.9)
Surgical Clinic	6 (28.6)	1 (7.1)	7 (20.0)
Pneumology Department	2 (9.5)	3 (21.4)	5 (14.2)
Gastro-Enterology Department	2 (9.5)	3 (21.4)	5 (14.2)
Cardiology/Pneumology	3 (14.3)	0	3 (8.6)
Paediatric Clinic	1 (4.8)	1 (7.1)	2 (5.7)
Dermatological Clinic	1 (4.8)	1 (7.1)	2 (5.7)
Cardiology/Internal Medicine	1 (4.8)	1 (7.1)	2 (5.7)
Trauma & Orthopaedics	0	1 (7.1)	1 (2.9)
**Ward**	**n (%)**		
ICU/IMC***	6 (28.6)	3 (21.4)	9 (25.7)
Normal Ward	15 (71.4)	11 (78.6)	26 (74.3)

*DQN – Domestically Qualified Nurses, **IQNs – Internationally Qualified Nurses, ***ICU/IMC – Intensive Care Unit/ Intermediate Care Unit

Qualitative content analysis produced one overarching key theme **Role of Nursing Management** and included five subthemes (Table 2). Exemplar quotes were used to illustrate meaning and themes identified during the analysis. Quotes were anonymised to protect the identity of participants. Illustrative quotations presented were translated into English and culturally adapted to maintain meaning by CR (a native German speaker fluent in English) and checked for accuracy by SB (a native English speaker fluent in German).

**Table 2:** Overview of themes and sub-themes

Role of Nursing Management	This theme describes the role of hospital management of hospitals in workplace integration of IQNs
**Subthemes**	**Definition**
Appropriate Placement	This subtheme describes the impact of (in)appropriate placement of IQNs on workplace integration from the perspective DQNs and IQNs.
Recruitment Process of IQNs	This subtheme includes factors related to the recruitment process of IQNs and impact on workplace integration from the perspective of DQNs and IQNs.
Leadership Support	This subtheme describes the impact of supportive leaders (or lack of) on workplace integration from the perspective of DQNs and IQNs.
Nursing Workforce Management	This subtheme describes the impact of nursing workforce management on workplace integration of IQNs from the perspective of DQNs and IQNs.
Additional Burden and Increased Workload	This subtheme describes perceived additional workload of DQNs due to workplace integration of IQNs as well as perceived workplace pressure of IQNs.

### Subtheme: appropriate placement of IQNs

DQNs highlighted that a key facilitator to successful workplace integration was appropriate placement based on previous clinical experience, nursing qualifications, and educational level. Appropriate placement was perceived as not only an essential factor for IQNs to develop professionally but also for DQNs to organise the integration process as efficiently as possible. DQNs mentioned that it was very challenging for IQNs, for example, to adapt to working in an intensive care setting (ICU) without previous ICU experience in combination with language barriers and cultural differences in nursing practice standards impeded ICU workplace integration. DQNs concluded that it would positively influence workplace integration if IQNs first worked on regular wards to improve their German language proficiency level, gained work experience in the German healthcare system, and got to know the hospital structures in a non-critical care environment. IQNs agreed with their domestically qualified colleagues. They explained that working in a new clinical setting significantly different from the one they had previously worked on at home was difficult and led to frustration and fear, these feelings negatively impacting workplace integration.

I would not let IQNs work directly in an intensive care unit. Let them work in a normal ward where the patients are not quite complex. Then they can first get to know the hospital, learn the language, and arrive here […] and also become familiar with the work processes here, […] learn ward management and time management. In my opinion, these are all tasks that can first be learned on a normal ward, and, as I said, the language can first be learned before they are sent to an intensive care unit where the work tasks are so much more extensive that there will probably be more problems and complications because you simply have to rely on every staff member that you have there to do the work well and then to take care of the patients. (DQN 6, Age 35).

[…] I have the fear I have the fear when I work on outpatient care unit or at the OR. I think that’s my weakness, yeah. […], but I like to work on a normal ward. (IQN 35, Age 29).

### Subtheme: recruitment process of IQNs

DQNs indicated that the recruitment process of IQNs played an essential role in successful integration to the workplace. They pointed out that too many IQNs starting at once made the integration process more difficult for the host nursing team, but this could be moderated depending on factors such as previous work experience or personal characteristics. Greater professional experience and internal personal motivation facilitated workplace integration. DQNs indicated that involving senior nurses who worked on the ward in the recruitment and integration process e.g., allowing them to express their opinion on the IQN’s suitability for the position was seen as a strategy to enhance workplace integration. IQNs, on the other hand, mentioned that starting together with other IQNs enhance workplace integration. They felt that peers’ support was valuable and helped them to overcome feelings such as homesickness, loneliness, and being an outsider.

Important for me is the right balance, for example, how many internationally qualified nurses and how many domestically qualified nurses are on the team, really good German-speaking nurses, […] that one can perhaps compensate for the other, also in terms of how we appear to the outside […]. (DQNs 29, 42).

We are the first group from the Philippines. I1:” Yes.” IQN 27: “Filipino, but I think it is better if others, for example, we have this second Filipino group, and I think it is better for them…. uh for the other group because we have… we have the other group support, we support, yeah.” I1: “Yeah, so the ones already there can support the ones coming, yes.” IQN 27: “Yes, exactly.” (IQN 27, Age 30).

DQNs highlighted that it was important to show IQNs that they were welcome and important team members, however, this was made more difficult if too many IQNs were started at once, as perceived workload increased with each additional IQN.

Yes, you must support the new employees. You must do something to make them feel comfortable. […] That’s why it’s so important that there are not too many at once, because otherwise you simply overburden the nursing team, you overburden yourself personally, and because they want to be noticed and taken seriously, and that’s a good thing. (DQNs 22, Age 40).

A few DQNs felt disadvantaged by the intense recruitment of IQNs. They mentioned that the funds spent on international recruitment could also be used for recruiting DQNs (e.g., targeting returners) and for improving the working conditions of nurses in Germany in general.

[…] they’re throwing a lot of money at recruitment of IQNs, but you can also give us more money, then maybe a lot more DQNs would come […].” (DQN 18, Age 30).

The importance of following ethical guidelines when recruiting nurses internationally was highlighted by both groups. Recruiting internationally qualified nurses exclusively from one country was seen as ethically unacceptable, as there was an equal need for qualified nurses the home countries. IQNs agreed with this. Yet individual choice and opportunities for professional advancement were recognised. IQNs stated that they emigrated to Germany for different reasons, mainly higher income, improved working conditions, job security and the opportunity to support their family in home country.

However, I believe that you have to do it in a targeted way and consider from which countries […] what I see critically is that the amount that is recruited, depending on which countries they come from, naturally also brings a disadvantage for the countries themselves at some point. (DQN 22, Age 40).

I think there are shortages of nurses all over the world and I think [migration of nurses] that’s good because we can exchange interculturally, we can learn something new from each other and we can change our… how can I say this? Our way of healthcare delivery if we work from for example international care, I think that’s good, but also in the Philippines there is a lot of staff shortage, but we don’t get enough money there, so we have to work in other countries.. (IQN 33, Age 29).

### Subtheme: leadership support

DQNs emphasised that it would positively influence workplace integration if the hospital management would regularly seek exchange with IQNs and DQNs to intervene early in case of workplace integration challenges. In addition, DQNs would appreciate it if their opinions and experiences regarding the integration of IQNs were recognised especially being given an opportunity to participate in decision-making related to workplace integration. Some DQNs indicated that nursing ward managers needed to be more actively involved in workplace integration and support IQNs’ professional development. DQNs concluded that workplace integration can only be successful if hospital management, nursing ward manager, and the nursing team collaborated effectively.

I have already spoken to the management about [the internationally qualified] staff. More attention could be paid to the [domestically qualified] staff when I say ‘but he/she [the IQNs] can actually do it’, that they [the management] consider it more and don’t say ‘no, he/she needs half a year of training’. There is too little flexibility. You have people who can already do a lot, why don’t I just let them? (DQNs 7, Age 38).

Yes, but otherwise nothing is done. And from the ward management, from some people, it doesn’t matter because they see that the work is being done, but of course no one looks over their fingers to see how you do it and it is just carried on like that. (DQNs 35, Age 30).

IQNs who participated in this study supported this view. They stated that they appreciated the support they received from their nursing ward managers. The majority of IQNs indicated that they felt supported and respected by their nursing leaders (regardless of the management level) most of the time. In addition, regular meetings were seen as helpful, especially in identifying and addressing problems early. In addition, IQNs highlighted that a supportive relationship with their nursing ward manager positively impacted on their integration process and their decision to stay in or leave their position, although some IQNs wished for more support and recognition during their integration process.

Yes, my ward manager has given me good support […] I have already said that I am very satisfied. […] Therefore, yes, I would like to stay here […]. What do I mean? One factor that a person like me, an employee, stay because the people or the… supervisor. (IQN 34, Age 28).

### Subtheme: nursing workforce management

DQNs saw advantages due to the recruitment and integration of the IQNs related to nursing workforce management. They mentioned that due to the migration of nurses, a sufficient number of nurses were available to fill roster gaps and deliver patient care. However, only some DQNs shared this opinion. Others believed that more needed to be done than recruiting IQNs alone to address the nursing workforce shortage in Germany. Nevertheless, a diverse nursing team was perceived as advantageous. For example, as the number of patients who did not speak German increased, IQNs could translate if they were from the same country of origin or spoke the same language as the patient. In addition, DQNs spoke of the great support they received from IQNs caring for patients and that they enjoyed working with the IQNs and valued their expertise. However, DQNs stressed that a constant lack of nursing staff had a negative impact on the integration process. They explained that IQNs often had to take responsibility for patients too early in the orientation period because of the lack of nursing staff. This situation increased the perceived workload for both groups and led to frustration. The IQNs supported this view and pointed out that staff shortages increased personal workload.

[…] Well, he is Italian [the IQNs], for example, if we have Italian patients and we do not understand them, and they do not understand us, he [the IQNs] is worth his weight in gold because he translates everything, […]. (DQN 18, Age 30).

Yes, and then there is also the difficulty that sometimes arises when there are few staff, IQNs may have to take on a lot of responsibility early on […], or do I have to check now when I am working on the ward and doing my area whether he or she is doing everything right in that area. […] that can be stressful for everyone. (DQN 8, Age 56).

So, that’s more for me…. means more responsibility for me and I have already worked alone without a responsible registered nurse. […] Nursing is a lot of responsibility for me […] I can say, “I can do it.” But I always have a lot of fear in my head, but I find that a lot of fear, but I can still control and I find that good, that when I’m afraid, I’m more careful and so, yeah. (IQN 33, Age 29).

### Subtheme: additional burden and increased workload

DQNs stated that workplace integration of IQNs promoted cultural learning and led to beneficial reflective questioning of their working methods. However, due to various constraints, the perceived workload of the DQNs increased. Some DQNs, for example, explained that information was lost in translation during handovers. As a result, DQNs had to find other sources of information to provide appropriate care to their patients, which increased their perceived workload. In addition, training and integrating IQNs required patience and time and this was perceived as challenging for the nursing team as a whole, particularly during intense shifts with high workloads. Furthermore, DQNs explained that one key challenge was determining whether their internationally qualified colleagues understood them correctly. They gave examples of IQNs completing nursing activities incorrectly because they did not understand what they were being asked to do or at least did not admit it. Some DQNs reported the need to double-check understanding, which placed an additional burden on them. IQNs indicated that workplace pressure had a negative impact on their integration. They described feeling overwhelmed due to caring for seriously ill patients, adapting to a work environment and learning a new language simultaneously.

[…] if I then hand someone over or have been handed over and it is then difficult to understand or I also find it difficult to understand what exactly is meant and queries can sometimes not be clarified and then even if no one else was there, no one else can answer the question, it then also makes it difficult or your own work process more protracted if you then have to get the information again from somewhere else. (DQN 16, Age 22)

[…] that they learn how to talk to the patients, that they also understand them and that they are also open and honest and say ‘Oh, I did not understand anything’, that is always a barrier. That they try to say ‘yes’ or even if you give an order or want them to do something, they say ‘yes’ and then you go and say ‘did you do it’ and then they did not understand, so that’s difficult for me.[…]. (DQNs 4, Age 35).

Yes, my work is a bit difficult, is complex, has to do a lot of talking to people and you need a lot of experience, and I don’t always manage what to do in my job […] (IQN 17, Age 35).

## Discussion

A critical factor identified by DQNs and IQNs was the role played by nursing management in the appropriate recruitment and placement of IQNs. Proactive support by leadership was considered beneficial by both groups of nurses. Constant nursing staff shortages and an increased workloads due to supervision of IQNs was perceived as hindrance to workplace integration by DQNs. Adaptation to a foreign work environment and high workloads overburdened IQNs and negatively impacted workplace integration.

Our study shows that inappropriate placement of IQNs had a negative impact on workplace integration due to the combination of insufficient professional experience related to the new clinical setting/placement and other challenges due to migration. In our study, this mismatch was expressed in particularly in the ICU environment by DQNs and IQNs. These study findings are consistent with Xiao et al. [37]. They conducted a study in two general hospitals in an Australian metropolitan city. They found that an unfamiliar work environments, nursing routines and clinical practices put an additional strain on IQNs. Moreover, DQNs’ workload increased due to the additional support they had to provide [37]. This additional burden and the perceived increased workload were also something DQNs in our study experienced. Brunero et al. investigated the experiences of nurses who migrated to Australia [38]. They concluded that if IQNs were able to find a clinical practice specialty or position similar to their previous position would enhance successful workplace integration, perception of competence and job satisfaction. According to Cowan et al. appropriate placement also reduces the likelihood of moving again [39]. Thus, to enhance workplace integration, nursing managers should ensure a good fit between their clinical area vacancies and the previous work experience of IQNs before hiring them.

The initial phase after arriving in the destination country is usually described as a time that can evoke negative feelings such as stress and anxiety [40]. Evidence suggests that peer support (people with shared experiences support each other) can be beneficial and prevent distress and negative feelings in IQNs [41–43]. IQNs in this study preferred to start working with other migrant nurses. They highlighted that the support they received by fellow IQNs was perceived as valuable and helped them to overcome feelings such as homesickness and being an outsider, confirming previous study findings. DQNs on the other hand stressed that the ratio of IQNs and DQNs per nursing staff should be assessed and an imbalance should be avoided. These suggests that the recruitment process and the initial phase after arrival in the destination country are crucial for IQNs as well as DQNs. Nurse managers should ensure that IQNs are supported by peers without overburdening the (host) nursing team due to an imbalance of DQNs and IQNs.

Findings also indicated that leadership support was a positive influence on the success of workplace integration of IQNs. DQNs, as well as IQNs, valued the support they received from their nursing manager. Nevertheless, room for improvement was identified in some aspects such as involving DQNs in the recruitment process and considering their opinion on internationally qualified applicants. The findings of the study by Brunton et al. [44] are somewhat different. Nurses across all cultures felt isolated and distressed due to a perceived lack of support from their management. The perceived unwillingness to address issues related to cultural differences within a multicultural team was especially stressful for nurses [44]. Our study suggests that support of the nursing management played a key role not only in improving and supporting the performance of the domestically and internationally qualified nurses but also in the well-being, satisfaction, and workplace integration of IQNs. Nurse managers should adapt different strategies to support their nursing teams such as regular meetings with ward nurses, domestically but also internationally qualified, and be open to improvement suggestions and perceived challenges.

The findings of our study confirm previous study findings that inadequate staffing negatively impacted on integration of IQNs and their job satisfaction [44]. Our study reveals that the level of despondency due to the constant shortage of qualified nursing staff and, thus, the increased workload during workplace integration led to frustration. This finding is consistent with Brunton et al. [44] who found that staff shortages and increased workloads impacted the well-being of both host nurses and migrant nurses. Nevertheless, our study showed that DQNs appreciated the positive impact on roster gaps by recruitment of IQNs to address existing workforce shortages. In addition, an unintended benefit to patients who come from several different cultural backgrounds, is that there is also a diverse nursing team. These results reinforce those of Brunton et al. [44] and Schilgen et al. [43]. Nurses qualified in New Zealand expressed the desire for greater workplace diversity [44]. German nurses joined their appreciation of the value of migrant nurses language or cultural interpreters [43]. This shows that recruiting IQNs nurses has benefits, as part of selected strategies to address workforce shortages. Nursing managers must consider various strategies and find ways to help their nursing teams coordinate workplace integration successfully despite resources constraints in the current healthcare environment.

Working conditions such as high workloads and work-related stress are associated with intention to leave the nursing profession [45, 46]. Work-related stress and high workloads were perceived by both IQNs and DQNs in our study. IQNs indicated that, in particular, working with critically ill patients (e.g. lifting heavy and immobile patients) and adapting to a foreign work environment simultaneously was perceived as hindrance to workplace integration. DQNs explained that supervising IQNs and supporting workplace integration increased their perceived workload. These study findings are consistent with Takenos [47] and Schilgen et al. [43]. Migrant nurses who participated in the study by Takeno stated that they were not accustomed to doing total patient care and were overwhelmed by paperwork [47]. Schilgen et al. found that migrant nurses associated working as a nurse in Germany with adequate working conditions but often worked in a very stressful environment [43, 48]. It is evident that workload and work-related stress impact on workplace integration and should not be underestimated. Thus, nurse managers should implement measures that reduce the additional burdens of DQNs and reduce perceived workload of IQNs where possible. Evidence suggests that introducing mentorship and preceptorship at ward level enhances workplace integration by taking the pressure of supervision of DQNs and supporting IQNs to adapt to the foreign work environment [40, 49, 50].

The migration of nurses has been increasing globally, particularly from lower-income countries with unstable healthcare systems to high-income countries [51]. In order to regulate the migration of health workers including nurses, the WHO developed the *Global Code of Practice on the International Recruitment of Health Personal* [52]. The WHO Global Code contains ethical principles that apply to the international recruitment of health personnel to strengthen the healthcare systems of developing countries. The aim of the WHO Global Code is among others to enhance more ethical recruitment on health workers to avoid recruitment in countries with acute shortages of health personal [52]. Although the WHO Global Code is not mandatory, Member States are encouraged to apply it. Both groups in our study highlighted the relevance of ethical guidelines when recruiting nurses internationally. According to a report by the International Council of Nurses (ICN), a member of the WHO Executive Board, large-scale nurse migration from low-income countries is due to active nurse recruitment by a small number of high-income countries among others Germany and the United Kingdom. The ICN indicates that losing highly skilled nurses to actively recruiting high-income countries is compromising the capacity of some countries to achieve health systems improvement and provide universal healthcare [53]. Thus, migration and workplace integration of IQNs must be based on ethical principles in order to ensure that patients receive healthcare globally.

### Strengths and limitations

The aim of this study was to explore how DQNs and IQNs experienced the role of nursing management during workplace integration. Although integration is a two-way process, many studies have investigated only the experiences of IQNs without considering those of the DQNs. A key strength of this study was the exploration of shared and/or contradictory experiences for both DQNs and IQNs included in the qualitative interview study. The selection of interviews as data collection method was seen as appropriate due to the explorative character of the research question. To minimise bias and reduce the risk of losing content, data analysis was guided by standardised methodological procedures, and the COREQ checklist was used to guide reporting qualitative findings [36].

A limitation that needs to be considered when interpreting the findings is that although experiences were relatively consistent in the study participants, findings may not be transferable outside the study setting. The results of this study may not apply to countries where migrant and host nurses speak the same language or share the same cultural or ethnic background. The same is true for countries with similar undergraduate nursing (or graduate entry programmes) education programmes to qualify nurses. National and regional factors may have influenced results and represent a limitation. Studies conducted elsewhere may produce different results even if a similar methodology were used. Although a higher number of nurses recruited from different hospitals and/or different regions may have led to more diverse results, at some point no new codes or themes were identified, which indicated that data saturation had been reached. In addition, nurses who chose voluntarily to participate may hold different thoughts and opinions compared to nurses that chose not to participate. For example, nurses who did not participate may evaluate the role of their nursing management completely differently. In addition, social desirability may have influenced participant responses, at least this potential cannot be excluded. Nevertheless, these findings give insights into how DQNs and IQNs perceive and experience the impact of nursing management on workplace integration into the German nursing workforce.

### Implications for practice

The findings of the study highlight the vital role of nurse managers in successful workplace integration of IQNs in the German nursing workforce. This study shows that appropriate placement of IQNs is a supportive factor. Nursing managers should be mindful of clinical specialty experiences IQNs have gained in their home country and where possible assign them to a similar ward within their new hospital based on this experience. The skillmix and ratio IQNs to DQNs per nursing staff should be assessed and an imbalance should be avoided. Appointing too many IQNs to a clinical area at once may have a negative impact on pace and effectiveness of integration and may increase the workload and work-related stress of the existing nursing team. Furthermore, the benefit of peer support for IQNs should not be underestimated. Compliance with international ethical guidelines (e.g., from the International Council of Nursing) in the recruitment of IQNs is advised. The choice of the country from which IQNs are recruited actively should therefore be based on international guidelines and regular re-evaluation by the nursing management. In addition, the active support of organisational leadership can have a positive effect on job satisfaction of the nursing team as a whole and thus influence workplace integration positively. Shared decision-making and recognition of both the challenges and the benefit experienced by DQNs during workplace integration of IQNs is crucial, especially considering the consequences of the shortage of qualified nurses. Although recruitment of IQNs is a strategy to address nursing workforce shortages in Germany, the impact of shortages of staff on the ward during the process of workplace integration cannot be underestimated. Integration of IQNs is accompanied by an additional burden for DQNs on top of an already high workload. In summary, nursing managers can positively influence the integration of IQNs into the German nursing workforce in many ways. These ways need to be explored in more depth in further qualitative and quantitative studies to improve the integration of IQNs and to facilitate their retention.

## Conclusion

The integration of IQNs into the workplace is complex and influenced by different factors. Nevertheless, nurse managers can positively influence the success of integration. The appropriate placement of IQNs based on their pervious clinical experiences seems to be a critical factor that has to be taken into account by nursing management in order to improve workplace integration of IQNs.

## Electronic supplementary material

Below is the link to the electronic supplementary material.

Supplementary Material 1

## Data Availability

The dataset that was generated and analysed during the study will not be made publicly available due to German data protection law.

## Abbreviations

COREQ: Consolidated Criteria for Reporting Qualitative Studies
DQNs: Domestically Qualified Nurses
ICN: International Council of Nurses
ICU: Intensive Care Unit
IMC: Intermediate Care Unit
IQNs: Internationally Qualified Nurses
WHO: World Health Organisation
