# Supplementary material for: Integrating internationally qualified nurses: a qualitative exploration of nurse managers’ influence from nurses’ experiences
Source: BMC Nurs. 2025 Feb 28;24:233. doi: 10.1186/s12912-025-02875-7 (PMC11869482; doi:10.1186/s12912-025-02875-7)
Supplement: Supplementary file 1 — Supplementary Material 1 [file 12912_2025_2875_MOESM1_ESM.docx]

**Integrating Internationally Qualified Nurses: A Qualitative Exploration of Nurse Managers' Influence from Nurses' Experiences**

Catharina Roth^1^, Amanda Breckner^1^, Katja Krug^1^, Cornelia Mahler^2^, Michel Wensing^1,3^, Sarah Berger^4^

1 Department of General Practice and Health Services Research, University Hospital Heidelberg, Heidelberg, Germany, Marsilius Arcades, West Tower, Im Neuenheimer Feld 130.3, 69120 Heidelberg, Germany

2 University Hospital Tuebingen, Department of Nursing Science, Hoppe-Seyler-Str. 9, 72076 Tuebingen, Germany

3 Heidelberg University, Ruprecht-Karls-University Heidelberg, Medical Faculty, Grabengasse 1, 60117 Heidelberg

4 Department of Nursing, University of Otago-Christchurch Campus, 2 Riccarton Ave, Christchurch 9140, New Zealand

| Catharina Roth B.Sc, MPH  **Corresponding author** | Dept. of General Practice and Health Services Research ,  University Hospital Heidelberg, Marsilius Arkaden, Turm West  Im Neuenheimer Feld 130.3  69120 Heidelberg  Germany  Phone: +49 6221 / 56 35736  Email: catharina.roth@med.uni-heidelberg.de |
| --- | --- |
| Amanda Breckner M.A. | Dept. of General Practice and Health Services Research ,  University Hospital Heidelberg, Marsilius Arkaden, Turm West  Im Neuenheimer Feld 130.3  D-69120 Heidelberg  Phone: +49 6223 / 56 34646  E-Mail: Amanda.Breckner@med.uni-heidelberg.de |
| Prof. Dr. Michel Wensing | Dept. of General Practice and Health Services Research ,  University Hospital Heidelberg, Marsilius Arkaden, Turm West  Im Neuenheimer Feld 130.3  69120 Heidelberg  Germany  Email: michel.wensing@med.uni-heidelberg.de |
| Prof. Dr. Cornelia Mahler | Dept. of Nursing Science  University Hospital Tuebingen  Hoppe-Seyler-Str. 9  72076 Tuebingen  Germany  Email: cornelia.mahler@med.uni-tuebingen.de |
| Dr. Katja Krug | Dept. of General Practice and Health Services Research ,  University Hospital Heidelberg, Marsilius Arkaden, Turm West  Im Neuenheimer Feld 130.3  69120 Heidelberg  Germany  E-Mail: katja.krug@med.uni-heidelberg.de |
| Dr. Sarah Berger | Department of Nursing  University of Otago-Christchurch Campus  2 Riccarton Ave  Christchurch 9140  New Zealand  Email: sarah.berger@cdhb.health.nz |

**Interview Guide used to conduct the interviews with Domestically Qualified nurses**

1. What do you think in general about the recruitment of internationally qualified nurses?
   - How does this affect delivery of patient care?
2. What do you think is particularly important during the orientation phase?
   - What would you do differently during the orientation phase?
3. To what extent is the ward manager on your ward involved in the orientation phase of internationally qualified nurses?
   - How could the nursing management (nursing manager and/or the ward manager) further contribute to facilitate orientation phase of internationally qualified nurses?
   - In your opinion, how can the workplace integration of the new internationally qualified nurses into the German nursing workforce be successful?
   - What other suggestions for improvement can you think of?
4. Do you see any challenges for internationally qualified nurses during delivery of patient care?
   - What challenges do you see for yourself?
5. How would you describe the teamwork with fellow nurses?
   - Can you think of any examples?
6. How would you describe the teamwork with other professional groups?
   - Can you think of any examples?
7. In your opinion, how does a qualification acquired in Germany differ from that of colleagues from other countries?
8. What could we in Germany learn from other countries in terms of nursing training?
9. In your opinion, how should internationally qualified nurses be prepared for working in Germany (apart from language)?
10. Practice standards and nursing activities can be diverse and very different.
    - How are these taught to internationally qualified nurses?
    - What training do you think would be helpful?
11. What can be done to address nursing shortages?
    - Salary is a big issue, but what else can be done to strengthen nursing?
12. Can you think of anything else you would like to mention that has not been addressed today?
13. If you could develop an integration concept for internationally qualified nurses, what wold you pay particular attention to in order to facilitate successful workplace integration?

**Interview Guide used to conduct the interviews with Internationally Qualified Nurses**

1. What are your general thoughts on the migration of internationally qualified nurses?
2. How would you describe the working conditions/working environment in your home country?
   - Are there any differences that you would particularly emphasize?
3. How have you/How did you experienced your orientation phase?
   - What would you do differently?
4. Do you have the impression that the nursing management (nursing manager and/or the ward manager) supports the orientation phase?
   - What would you like to see from your current nursing management (nursing manager and/or the ward manager)?
5. Do you have the impression that workplace integration is supported by the nursing management (nursing manager and/or the ward manager)?
   - What would you like to see from the nursing management (nursing manager and/or the ward manager)?
6. Can you think of measures that could be improved to enhance workplace integration?
   - What could the hospital do to help you integrate into the team successfully?
7. What was/is the greatest challenge for you?
8. What challenges do you see in delivery of patient care?
   - Do you feel up to the challenges?
   - Do you have the impression that you are treated fairly?
   - How would you describe working with patients?
9. How would you describe the teamwork with fellow nurses?
   - How would you describe the teamwork with other professional groups?
   - Can you think of any examples?
10. How do you experience the differences in nursing qualifications or education?
11. Are there any special training courses that you would like to take in order to master everyday work on the ward?
    - Which training courses do you find helpful?
12. What can be done to address nursing shortages?
    - Salary is a big issue, but what else can be done to strengthen nursing?
13. Can you think of anything else you would like to mention that has not been addressed today?
14. If you could develop an integration concept for internationally qualified nurses, what wold you pay particular attention to in order to facilitate successful workplace integration?
